# A First Randomized Eight-Week Multidisciplinary Telerehabilitation Study for the Post-COVID-19 Condition: Improvements in Health- and Pain-Related Parameters

**DOI:** 10.3390/jcm14020486

**Published:** 2025-01-14

**Authors:** Indre Bileviciute-Ljungar, Agneta Apelman, Lena Braconier, Sara Östhols, Jan-Rickard Norrefalk, Kristian Borg

**Affiliations:** 1 Department of Clinical Sciences, Karolinska Institute, Danderyd University Hospital, 18288 Stockholm, Sweden; norrefalk@hotmail.com (J.-R.N.);; 2 Multidisciplinary Pain Clinic, Capio St. Göran Hospital, 11219 Stockholm, Sweden; agneta.apelman@capiostgoran.se (A.A.);; 3 Department of Physical and Rehabilitation Medicine, Danderyd University Hospital, 18288 Stockholm, Sweden

**Keywords:** post COVID-19 condition, International Classification of Functioning and Disability (ICF), telerehabilitation, multidisciplinary, chronic pain, SF-36, anxiety, depression

## Abstract

**Background/Objectives:** This study investigates the effects of multidisciplinary telerehabilitation for the post-COVID-19 condition. **Methods**: Recruitment was announced during 2021/22 through the COVID patient organisation in Sweden. The key inclusion criteria were persistent symptoms and functional impairments longer than 12 weeks after an acute SARS-CoV-2 infection, a stable health condition, and satisfactory skills to use the Internet. Participants were randomised into a rehabilitation group or a waiting list. Telerehabilitation was performed by a multidisciplinary team. Measured outcomes included health- and pain-related parameters and pain-related disability after eight weeks and when followed up after six months. **Results**: In total, 164 participants registered for the study. Of them, 67 participated in an eight-week group telerehabilitation programme (mean age 43, 78% women) and 42 stayed on a waiting list (mean age 47, 88% women) after eight weeks. Sixty participants from the rehabilitation group and 21 from the waiting list completed the data at the six-month follow-up. The results show greater improvements in both health- and pain-related parameters within the telerehabilitation group after eight weeks and six months than within the waiting list group (Cohen’s d effect size varied between moderate and large). However, no statistically significant differences were found between the groups, except participants being at a lower risk of anxiety measured based on the Hospital Anxiety and Depression Scale after eight weeks of telerehabilitation compared to those on the waiting list. **Conclusions**: The results indicate that multidisciplinary group telerehabilitation is beneficial for improving health- and pain-related parameters in people suffering from the post-COVID-19 condition and should be further developed and adapted for similar conditions, such as chronic pain, fatigue, etc.

## 1. Introduction

Pain has been reported as a frequently found symptom in the post-COVID-19 condition (long COVID), a clinical case definition established by the World Health Organization (WHO) in September 2021 [1]. For example, Oguz-Akarsu et al. investigated a cohort of 222 subjects (mean age 42 years, 48% females), 37.5% of whom were not hospitalised, interviewed between 1.5 and 3 months after a positive PCR test [2]. Among participants, 72% reported pain [2]. Chronic new-onset pain after SARS-CoV-2 infection has been reported in approximately 43% of 226 hospitalised patients, with a prevalence of 65% for new-onset pain, while a control group reported 11% (73 participants hospitalised for reasons unrelated to COVID) [3]. However, the overall number of symptoms reported by hospitalised and non-hospitalised participants was the same at 2 years after acute SARS-CoV-2 infection, with fatigue (approximately 45%), pain (approximately 32%), and loss of memory (approximately 18%) among retrospectively evaluated 668 participants using telephone interviews [4]. Although, the acute infection was mild, the number of persons diagnosed with the post-COVID-19 condition afterwards is large compared with those who had a serious acute infection. In Sweden, an epidemiological study of the ICD-10 diagnosis of the post-COVID-19 condition showed a prevalence of approximately 2% among 4.1 million inhabitants [5]. A major part of the population (70%) had a mild acute infection and was not hospitalised.

According to the results of our recent study of 100 participants after mild SARS-CoV-2 infection, approximately 91% had new-onset pain symptoms and 40% were assessed to fulfil the 2016 fibromyalgia criteria when assessed longer than 12 weeks after infection [6]. However, our study also showed that pain is not a major concern among the symptoms affecting functioning and the level of disability [7]: fatigue, neurocognitive complaints, and cardiopulmonary symptoms were scored as having a greater impact in terms of impairing body functions and activities [7]. The impact of pain in the post-COVID-19 condition also varies depending on other comorbidities [4,8], previous pain conditions [3], and sociodemographic variables [9,10]. It is therefore difficult to compare pain as a symptom between different populations in terms of the impact of pain on functioning and disability.

Telerehabilitation increased during COVID pandemics for different health conditions [11], and overall results seem to be similar compared to conventional therapy [12,13]. However, the implementation of it and adapted evaluation toolkits need further development [14]. The Telerehab Toolkit has been developed for practitioners and patients and is available online [15]. However, the use of the International Classification of Functioning, Disability and Health (ICF) might be a complement in evaluating the effects of telerehabilitation. The ICF provides a complete and standardised picture of an individual’s functioning, activity, and participation, approved by the WHO in 2001 [16]. Even though the ICF is a well-known tool to evaluate the body’s functioning and activities/participation, the reports on using it for the post-COVID-19 condition are few [7,17].

The aim of this study was to investigate the effects of multidisciplinary group telerehabilitation on health- and pain-associated symptoms, including pain-related ICF categories, for participants with the post-COVID-19 condition after mild infection. The hypotheses were as follows: (1) multidisciplinary eight-week group telerehabilitation will significantly improve health- and pain-related symptoms and functions as compared to the waiting list; and (2) the positive effects of multidisciplinary group telerehabilitation will last even at six months of follow-up. Primary outcomes were health- and pain-related symptoms, including ICF categories related to pain. Secondary outcomes were anxiety and depression.

## 2. Materials and Methods

### 2.1. Participants

The study protocol has already been published [7]. In brief, recruitment took part via Facebook sites and “long COVID” stakeholder organisations in Sweden, gathering people with the post-COVID-19 condition. To be able to participate, the applicants were obliged to sign an agreement, complete all questionnaires, and perform two physical tests with a pulse oximeter on their own hand. Afterwards, the randomisation was performed using an Internet program at RANDOM.ORG., accessed on 5 May 2021, 27 August 2021, and 28 April 2022.

Inclusion criteria were as follows: (a) presence of acute COVID-19 infection supported by medical history and/or a positive PCR test for the COVID-19 virus and/or a positive immunoglobulin response; (b) age between 18 and 70 years; (c) significantly reduced levels of functioning and activity/participation in daily life after infection (appreciated 50% reduction); (d) remaining symptoms started after acute infection and persisted for at least 12 weeks when entering the study; (e) comorbidities and/or new post-infection symptoms were evaluated and managed by primary health care; and (d) skills to use the Internet when completing questionnaires and participating in a rehabilitation programme in a group over an eight-week period. Exclusion criteria were as follows: (a) unclear onset of symptoms in relation to COVID-19 (for example, stress factors, untreated/unstable psychiatric, psychological and somatic conditions in conjunction with or prior to a COVID-19 infection); (b) overuse/abuse of alcohol or psychotropic substances; (c) unstable somatic or psychological conditions requiring appropriate treatments (e.g., lung, heart, kidney disorders, hypothyroidism, psychiatric conditions, including suicidality, etc.); and (d) ongoing treatment of psychiatric or medical conditions, which may interfere with the rehabilitation (for example psychotherapy, intermittent pharmacological treatment during the study period).

For registration to the study, the participant had to log into the BASS, which is an online data platform provided by the eHealth Core Facility at Karolinska Institutet. A link to access information and a consent document was provided at the first stage. The consent document was signed using an electronic national signature (BankID) and submitted to BASS at the second stage. Thereafter, the participant got a link to complete and submit questionnaires to the BASS. Data for the BASS was gathered by sending a link at the start of the study, after eight weeks and at six months of follow-up for those participants who participated in the study or as indicated in the ethical permission. Due to the lack of personnel during pandemics (2021–2022), it was not possible to conduct the study as it was planned from the beginning. The waiting list was supposed to enter rehabilitation after six months, providing data both for the six-month follow-up and for the start of the next rehabilitation group. This resulted in the absence of the six-month follow-up data for the first two groups as was originally planned. A latter adjustment to the ethical permission allowed for the data to be collected for the waiting list per see (third group). This resulted in a lesser amount of data for the waiting list at the six-month follow-up.

### 2.2. Questionnaires

The 36-Item Short Form Health Survey (SF-36) is a widely used questionnaire to evaluate health-related quality of life [18,19]. The 36 items in the questionnaire consists of eight subscales: physical functioning, role limitations caused by physical problems, bodily pain, general health, energy/vitality, social functioning, role limitations caused by emotional problems, and mental health. Additionally, subscales for physical and mental cumulative scores were calculated for this study.

The Hospital Anxiety and Depression Scale (HADS) is used to measure levels of anxiety and depression [20]. Scores for both parameters range from 0 to 21. A score of 11 or above is considered abnormal and may indicate clinically significant anxiety or depression.

The Patient Health Questionnaire-9 (PHQ-9) is a questionnaire to assess depressive symptom severity [21]. PHQ-9 has nine-items, which are rated from 0 to 3 (total scores ranging from 0 to 27). The standard cut-off score for screening to identify possible major depression is 10 or above [22].

The Generalised Anxiety Disorder-7 Scale (GAD-7) is a seven-item questionnaire to assess anxiety symptom severity. Self-reported items are rated 0 to 3, with total scores ranging from 0 to 21. The standard cut-off score for screening to identify possible anxiety disorder is recommended as 10 or above [23].

Pain intensity during the last week was measured using a numeric scale (0 = “no pain” and 10 = “worst imaginable pain”, for a total of 11 points). Pain was rated based on the presence or absence of pain in 36 body sites, 18 on either side: head and/or face, throat and/or neck, shoulder, upper arm, elbow, lower arm, hand, front of the chest, side of the chest, abdomen, sexual organs, upper part of the spine (thoracic spine), lower part of the spine (lower back), hip and/or seat, thigh, knee, lower leg, feet. The last question was which body site was most painful, with the following alternatives: head/face, throat/neck, shoulder and/or arm, chest, abdomen, sexual organs, upper part of the spine (thoracic spine), lower part of the spine (lower back), hip and/or seat, legs, or “main worst region varies”.

The Functional Compass COVID-19 Questionnaire is developed from the Functional Barometer [24] with ICF categories in purpose to cover theoretically predicted disability after a COVID infection [16]. Here, we report eight body functions related to pain and based on the same ICF category, b280 “Sensation of pain” ((four of which have been previously reported [7], and four body functions related to skin sensitivity, three of which based on ICF b270, “Sensory functions related to temperature and other stimuli”, and one on b1564, “Tactile perception” [16])). All items were assessed on a verbal descriptor scale, the same as the ICF qualifier. All categories were rated on a scale of 0–4, defined as no (0), light (1), moderate (2), severe (3), and total (4) problems/impairments [16].

### 2.3. Rehabilitation Programme

The detailed content of the multidisciplinary group telerehabilitation programme has been published elsewhere [7]. In brief, the telerehabilitation programme was given in groups for two hours a day, three days a week over a period of eight weeks on the Teams platform. Participants were also asked to exercise for at least 3 h a week by themselves and report the results in the ExorLive app (https://www.exorlive.com/uk, accessed from 1 May 2021). Team members also offered all participants weekly 20 min individual sessions on Teams throughout the eight-week programme, using the “Microsoft Teams” platform (https://www.microsoft.com/en-us/microsoft-teams/group-chat-software, version “classic”, accessed from 1 May 2021).

Regarding dealing with pain in the programme, a clinician gave one psychoeducational lecture on pain related to the COVID infection, one session on sleep disturbances and one session on chest pain, the latter included in a psychoeducational lecture on cardiopulmonary symptoms. Post-exertional malaise and postural orthostatic tachycardia syndrome were discussed during the clinician’s session on central and peripheral nervous system disturbances. The individual management of long COVID symptoms, including pain, was followed up through individual appointments with team members and by using the ExorLive application. Participants were able to use training exercises in the application and communicate directly with a physiotherapist. Individual feedback was also offered by a psychologist and occupational therapist during appointments. The clinician (pain specialist) evaluated the severity of the participant’s symptoms and indirectly recommended prescriptions for tricyclics or antiepileptics, depending on the symptoms, by the primary care doctor. Participants were suggested to contact their doctors at the primary healthcare system.

The study has been registered on ClinicalTrials.gov (identifier: NCT04961333) and approved by the Swedish Ethical Review Authority (ref. no. 2020-07216).

### 2.4. Statistics

A power analysis was performed for pain intensity accepting that a minimum change in pain based on NRS is 1.74 [25,26]. The type I error was set at 0.05 and the power of the statistics at 0.80. The number of a sample was estimated to include 80 participants. The power analysis for SF-36 with a minimum significant change of 10 in the subscales estimated approximately 63 participants in each group.

Descriptive statistics for nominal data are presented as the number of participants and percentage per group. Statistics for ICF qualifiers are presented as the median (range from 0 to 4) and percentage for every category. Quantitative data are presented as the mean, standard deviation, and range. To compare the rehabilitation group with the waiting list, the Chi-Square or Mann–Whitney tests were used for nominal/qualitative data, with an independent *t*-test for quantitative data. A Wilcoxon test was used to calculate comparisons within the groups between two-time measurements, while a non-parametric Friedman test was used for three-time measurements.

To obtain Cohen’s d effect size within and between the groups, a paired sample test and one-way ANOVA was applied to SF-36, HADS, GAD-9, and PHQ-9.

A general linear model was used to identify the role of the age, gender, and group measured over time. Time was chosen as a within-subjects factor, grouped as a between-subjects factor, and age was a continuous covariate when gender was a nominal covariate. The general linear model was applied for pain intensity, with SF-36 categories for Physical and Mental Cumulative Scores, as well as HADS, GAD-7, and PHQ-9.

Statistical Package for the Social Sciences (SPSS) version 28 was used for analyses.

## 3. Results

### 3.1. Sociodemographic Data and Comorbidities

**Figure 1:**
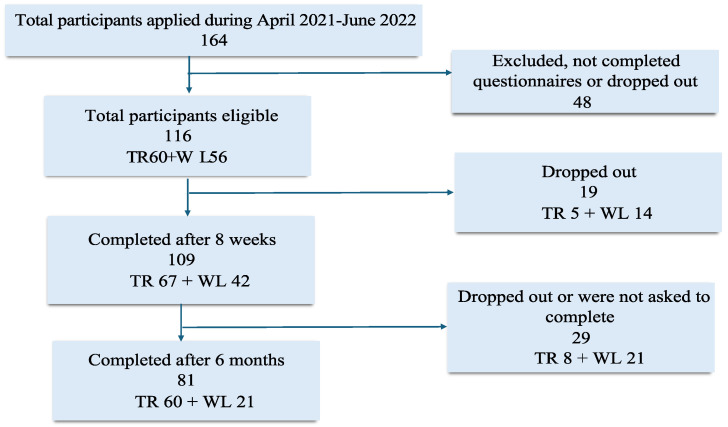
Flow chart of the study protocol. Abbreviations: TR = telerehabilitation group; WL = waiting list.

The recruitment and randomisation flow-chart is presented in Figure 1 (also previously reported). Briefly, 116 participants participated and 109 participants completed the questionnaires after eight weeks, 67 from the telerehabilitation group and 42 from the waiting list at the eight-week follow-up. The mean age of participants was 45 years, and most of them were females (82% women) and born in Sweden (90%). Approximately 70% had a higher education, and over 50% were working at the start of the study, while approximately 35% were on full- or part-time sick leave. No statistical differences were found between the groups, including (1) the symptom duration ((51 (SD 27) and 58 (SD 31) weeks, rehabilitation and waiting list respectively)), (2) performance of the SARS-CoV-2 PCR test (59% and 41% rehabilitation and waiting list, respectively) and a positive PCR reaction (63% and 38% rehabilitation and waiting list, respectively), (3) an antibody test (60% and 40% rehabilitation and waiting list, respectively) and a positive antibody result (52% and 48% rehabilitation and waiting list, respectively), (4) hospitalisation (10% and 14% rehabilitation and waiting list, respectively), and (5) comorbidities and medication before the acute SARS-CoV-2 infection (total comorbidities 0–4/5 and total medication 0–7/8 per each group). No differences were found between the groups for all sociodemographic parameters measured, except for age, in the pooled final cohorts showing that participants on the waiting list were 3 and 7 years older than the telerehabilitation group at the eight-week and six-month follow-up, respectively (independent sample test, results not presented).

### 3.2. Health-Related Parameters

**Figure 2:**
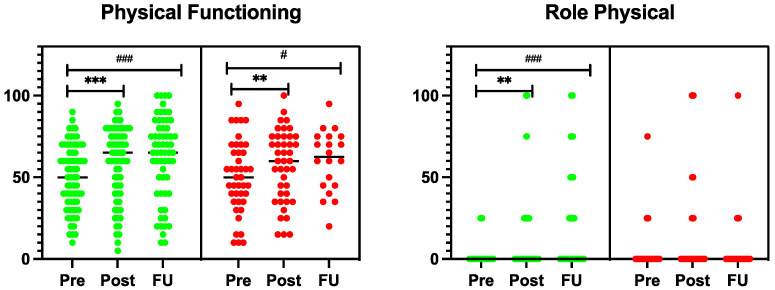

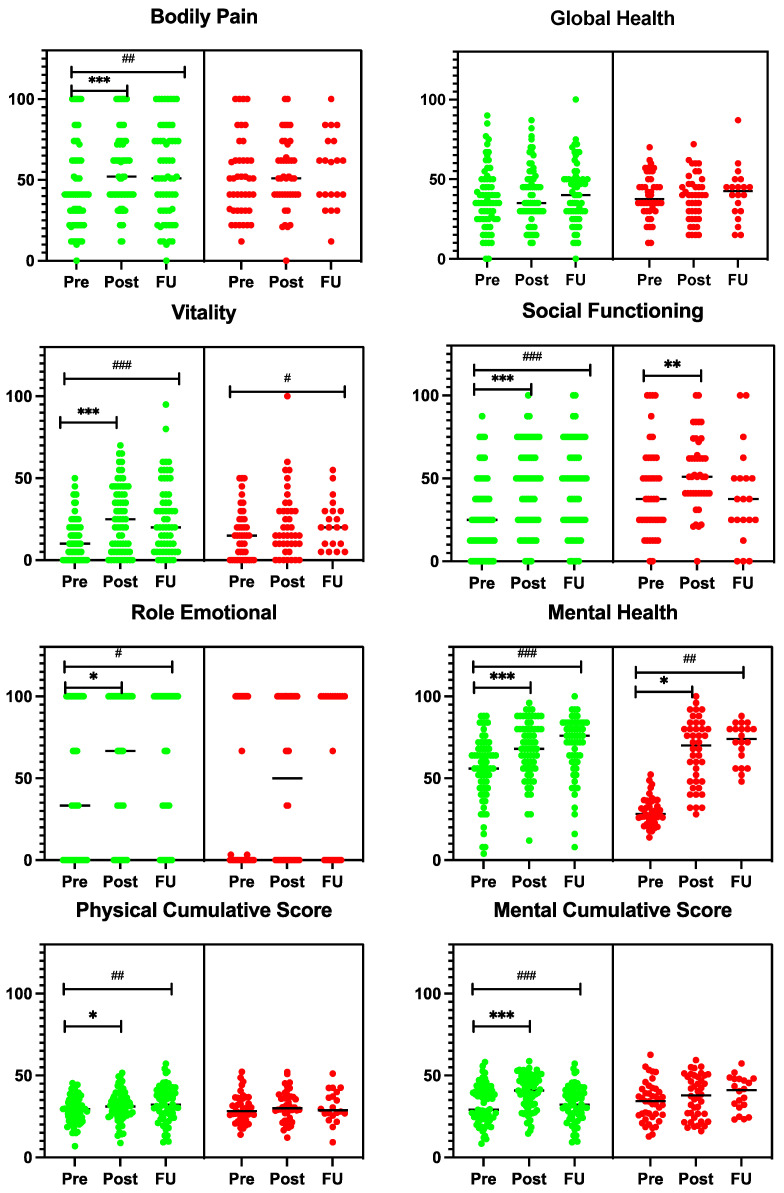
The results of SF-36 are presented as individual values. A dark line between the values indicates the median value in every group. A green colour represents the telerehabilitation group and red, the waiting list participants. * indicates changes within the group between the start (Pre) and end of the rehabilitation period (Post), * *p* < 0.05, ** *p* < 0.01, and *** *p* < 0.001. # indicates changes within the group between the start (Pre) and six-month follow-up (FU), # *p* < 0.05, ## *p* < 0.01, and ### *p* < 0.001.

For health-related parameters, absolute values for SF-36 were analysed with no differences between the groups (one-way ANOVA). To calculate Cohen’s d effect sizes, a paired sample *t*-test was used, showing an improvement within the telerehabilitation group for physical functioning, role limitations caused by physical problems, bodily pain, energy/vitality, social functioning, role limitations caused by emotional problems, mental health, and subscales for physical and mental cumulative scores, with a Cohen’s d of physical functioning = 0.5, role physical = 0.4, bodily pain = 0.5, vitality = 0.7, social functioning = 0.6, role emotional = 0.3, mental health = 0.9, physical cumulative score = 0.3 and mental cumulative score = 0.7 after eight weeks and physical functioning = 0.6, role physical = 0.5, bodily pain = 0.4, vitality = 0.6, social functioning = 0.6, role emotional = 0.3, mental health = 0.9, physical cumulative score = 0.4, and mental cumulative score = 0.6 at the six-month follow-up (Figure 2). Participants on the waiting list showed significant improvements in physical functioning, social functioning, and mental health, with a Cohen’s d of 0.5, 0.4, and 0.4, respectively, after eight weeks, and in physical functioning, vitality, and mental health with a Cohen’s d of 0.6, 0.6, and 0.8, respectively, after six months (Figure 2). Neither group showed any significant improvement in general health.

### 3.3. Pain-Related Parameters

#### 3.3.1. Pain Intensity

Pain intensity for the telerehabilitation group was 4.6 at the start, 3.9 after the eight-week programme, and 4.2 when followed up after six months. There was as a small but significant reduction in pain intensity after telerehabilitation, with a Cohen’s d of 0.3 (*p* < 0.026, paired samples test, N = 67) and a small but non-significant reduction (0.22, *p* = 0.087, paired samples test, N = 60) after six months. The corresponding results for the waiting list were 3.9 at the start, 3.9 after eight weeks, and 4.1 when followed up after six months. Cohen’s d indicated non-significant small size effects within the waiting list after eight weeks (0.02, *p* = 0.9, N = 42) and after six months (0.2, *p* = 0.36, N = 21). No differences were found between the groups (one-way ANOVA).

#### 3.3.2. IASP Pain Sites

An analysis of IASP 36 pain sites shows that the majority of participants indicated pain in the head, throat/neck, shoulder, and thorax, rather than in the thoracic or lower spine and extremities (Supplemental Table S1). At the start, more participants in the waiting list reported pain in the left part of the head (72% vs. 62%, *p* < 0.01, Chi-Square test, N = 109), left elbow (16% vs. 11%, *p* < 0.037, Chi-Square test, N = 109), and right elbow (19% vs. 15%, *p* < 0.033, Chi-Square test, N = 109) and tended to report more pain in the right part of the head (72% vs. 66%, *p* < 0.089, Chi-Square test, N = 109). After eight weeks, participants in the telerehabilitation group reported less pain in the lower part of both legs (left 13% vs. 19%, *p* < 0.046, Chi-Square test, N = 109). No differences between the groups were apparent when followed up after six months. Repeated non-parametric measurements (N = 60) showed that the telerehabilitation group reported a reduced number of the following pain sites: right upper arm (*p* < 0.018), left and right forearms (*p* < 0.018 and *p* < 0.038, respectively), and right thigh (*p* < 0.03), based on the Friedman test. No statistically significant changes were seen in the waiting list group.

#### 3.3.3. ICF Pain-Related Qualifiers

**Figure 3:**
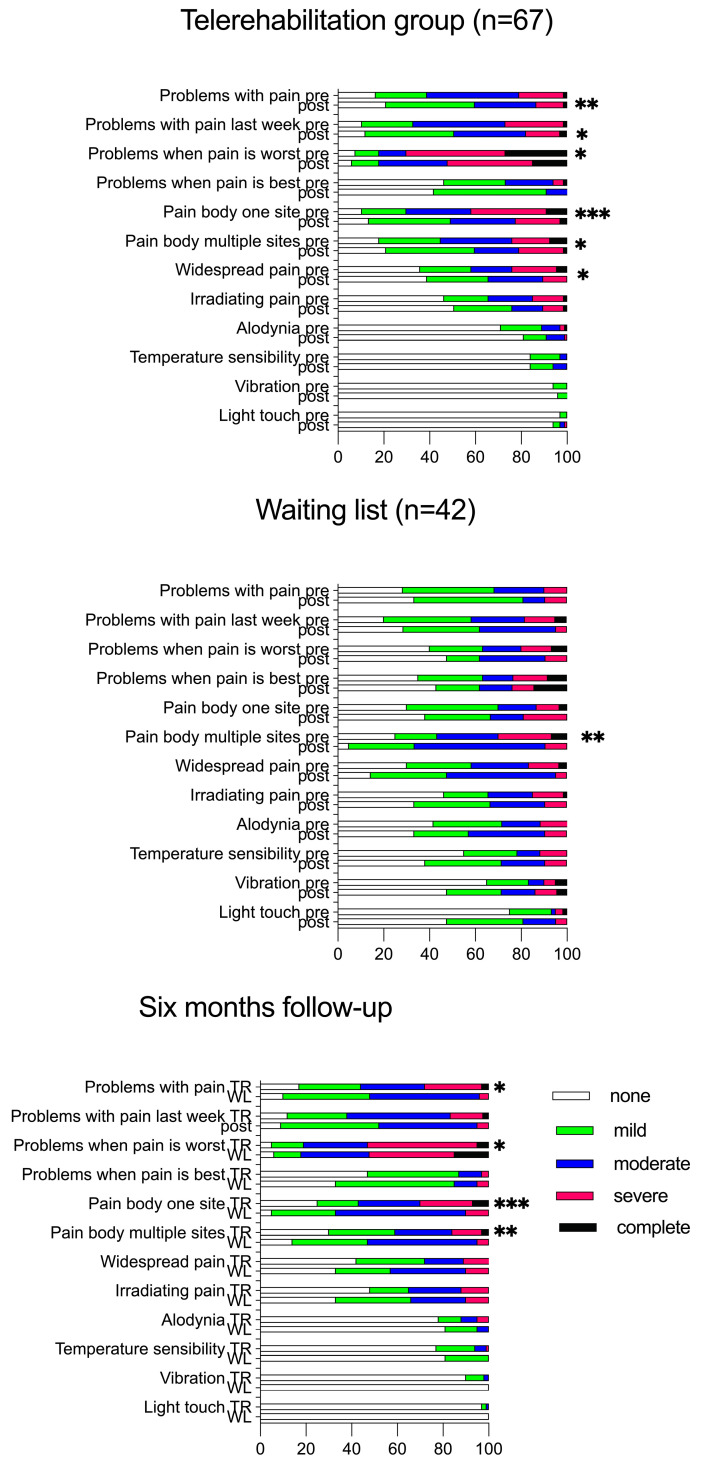
ICF b-categories are presented as the percentage at the start (pre), after eight weeks (post), and at the six-month follow-up for participants in the telerehabilitation group and waiting list. A non-parametric Wilcoxon signed rank and Friedman tests within the groups were used for eight-week and six-month follow-ups, respectively. * *p* < 0.05, ** *p* < 0.001, and *** *p* < 0.001. For the six-month follow-up, n = 60 for telerehabilitation group (TR) and n = 21 for the waiting list (WL).

Twelve pain-related ICF b-categories were analysed, revealing no differences between the groups after either eight weeks or six months (Mann–Whitney non-parametric test). The results of ICF qualifiers are presented in Figure 2, which shows that participants in the telerehabilitation group reported a greater improvement in pain-related ICF categories: “problems with pain”, “problems with pain last week”, “problems when pain is worst”, “pain body one site”, “pain body multiple sites”, and “widespread pain” after the eight-week telerehabilitation programme. Participants on the waiting list reported an improvement in pain in multiple body sites after eight weeks. When followed up after six months, the application of a non-parametric analysis for repeated measurements within each group showed improvements in participants in the telerehabilitation group in terms of “problems with pain”, “problems when pain is worst”, “pain body one site”, and “pain body multiple sites”, while the waiting list group showed no improvements. Few study participants reported impairments in skin sensitivity functions (Figure 3).

### 3.4. Secondary Outcomes (HADS, GAD-7, and PHQ-9)

For psychological parameters, HADS (domains for anxiety and depression), GAD-7, and PHQ-9 were analysed. No statistically significant differences between the groups were found in absolute values of HADS, GAD-7, or PHQ-9 at the start after eight weeks (, or when followed up after six months (one-way ANOVA, *p* > 0.05). When comparing the number of participants with pathological values, the number of participants with a risk of anxiety significantly decreased by 10% in the telerehabilitation group compared to the waiting list at eight weeks. There were no significant disparities between the groups within the other parameters (Table 1).

To calculate Cohen’s d effect sizes, a paired sample *t*-test was used, showing a decrease in anxiety and depression (measured using HADS) within the telerehabilitation group, with effect sizes of 0.4 (*p* < 0.01) and 0.6–0.7 (*p* < 0.001) at eight weeks and the six-month follow-up, respectively. For the waiting list, only scores in depression were reduced significantly, with a Cohen’s d effect size of between 0.5 and 0.6 at both eight weeks (*p* < 0.01) and six months (*p* < 0.05).

For GAD-7, the telerehabilitation group improved after both eight weeks and six months, with a Cohen’s d effect size of 0.4 (*p* < 0.01) and 0.3 (*p* < 0.05), respectively, while the waiting list group did not show any improvement. For PHQ-9, the telerehabilitation group improved both at eight weeks and six months, with a Cohen’s d effect size of 0.8 (*p* < 0.001) and 0.5 (*p* < 0.001), respectively, while the waiting list showed improvements when followed up after six months, with a Cohen’s d effect size of 0.6 (*p* < 0.05).

### 3.5. Impact of Age and Gender on the Results

General linear models for pain intensity, SF-36 categories for Physical and Mental Cumulative Scores, and for HADS, GAD-7, and PHQ-9 were analysed to understand the impact of the intervention, gender, and age. Results showed gender having an impact on pain intensity (N = 60 and 24, F = 8.1, *p* < 0.01) and the physical cumulative score (N = 60 and 24, F = 19.4, *p* < 0.001) but not for mental cumulative score, HADS, GAD-7, and PHQ-9, when the three-time measurement was chosen. When the two-time measurement was chosen (pre- and post-rehabilitation results only), both pain intensity (N = 67 and 42, F = 9.5, *p* < 0.01) and the physical cumulative score (N = 67 and 42, F = 17.1, *p* < 0.001), as well as the mental cumulative score (N = 67 and 42, F = 6.7, *p* < 0.05), were influenced by gender but not by age or intervention.

## 4. Discussion

This study demonstrates that an eight-week multidisciplinary telerehabilitation programme had positive outcomes for people with long COVID in health- and pain-related parameters. That said, no significant differences were found between the groups, whether directly after telerehabilitation or when followed up six months later, except for a reduction in anxiety among the telerehabilitation group after completing the eight-week programme.

Telerehabilitation improved health-related quality of life as measured by the SF-36 to a larger extent compared to the waiting list. However, participants on the waiting list also scored improvements in physical and social functioning, vitality, and mental health with no differences between the groups. While the study focuses on health- and pain-related characteristics, it also includes psychological questionnaires, relevant for chronic pain conditions. Among those on the waiting list, only depression scores measured using HADS improved after eight weeks and six months. Such spontaneous improvements in mood might be explained by the increased awareness of long COVID among healthcare providers and in society as a whole over the course of the pandemic and the availability of vaccines from 2021 onwards. Others have also reported improved health-related parameters measured using GAD-7, PHQ-9, and SF-36 in long COVID after rehabilitation; however, trials were not randomised [27,28].

This is the first study of multidisciplinary group telerehabilitation to analyse health- and pain-related parameters for people with long COVID. We have recently reported some of the results from the study of multidisciplinary group telerehabilitation for patients with long COVID, including measurements of the health-related quality of life, breathing scales, and 38 ICF items for body functions (22 b-categories) and activities and participation (16 d-categories) [7]. The results of the present report are in line with previously reported improvements in many ICF b- and d-categories, showing general improvements in the telerehabilitation group as compared to the waiting list after eight weeks of telerehabilitation, even if the differences were few [7]. Although the results showed improvements in both the rehabilitation group and the waiting list over time, including the six month follow-up, the telerehabilitation group showed a greater improvement in all measured outcomes compared to the waiting list [7].

The results indicate that, while the pain intensity was relatively low, many participants reported pain in multiple body sites and particularly in the head, neck/throat, thorax, spine, and extremities. In the telerehabilitation group, both pain intensity and pain sites decreased, although the effect sizes for pain intensity were low, and only a few pain sites were reduced within the study period. Despite the relatively low reduction in pain intensity and painful sites, the psychological parameters for anxiety and depression also improved to a larger extent in the telerehabilitation group as compared to the waiting list. Statistical significance for a reduced risk of anxiety according to HADS was obtained after 8 weeks as compared to the waiting list. However, no differences were found in GAD-7 and PHQ-9 between the groups.

The SARS-CoV-2 pandemic increased the use of digital technology in healthcare [29], especially in pulmonary rehabilitation [30]. Satisfaction using telerehabilitation during pandemics was reported by participants and physical therapists according to a qualitative study in Kuwait [31]. A literature review on the use of telerehabilitation for long COVID reports found only a few studies of variable quality and uncertain outcomes [32]. Many studies report unimodal interventions, such as digital physiotherapy and often with no waiting control list, creating difficulties to evaluate spontaneous improvements in the post-COVID-19 condition [33,34,35]. Recently, a randomised controlled study reported greater improvements in physical activities/performance, health-related quality of life, and dyspnoea in patients with fatigue and dyspnoea after a seven-week mixed online program [36]. However, the study was performed in the primary health setting and by physical and occupational therapists excluding patients with other long COVID symptoms and those having more than two comorbidities [36]. Therefore, it is unclear if patients fulfilled the criteria for the post-COVID-19 condition according to the WHO [1]. On the other hand, a systematic review and meta-analysis of rehabilitation interventions for adults with long COVID reports improvements in functional exercise capacity, dyspnoea, and quality of life compared to the current standard care [37]. The present study is the first to present health- and pain-related parameters after telerehabilitation for the post-COVID-19 condition, and it clearly shows that multidisciplinary group telerehabilitation improves parameters related to the health-related quality of life, pain, and psychological mood.

Participants in the present cohort were dominated by middle-aged and highly educated women after a mild SARS-CoV-2 infection. Several studies have found that younger and middle-aged women usually dominate in long COVID-19 studies, in particular after a mild acute infection [38]. The randomisation of study participants is important to minimise differences in sociodemographic parameters and initial parameters. Results show that gender was a covariate having an impact on pain intensity and physical/mental functioning at the 8-week follow-up although the mean age of the waiting list was higher than that of the rehabilitation group after the eight-week and the six-month follow-up. In a previous study using the ICF domains of the same cohort, we demonstrated that age was mainly associated with improvements in heart functions and remunerative employment [7].

The strengths of the study were as follows: randomisation, three separate rounds of recruitment for the study, individual appointments allowing to adapt participation and individual goals during the telerehabilitation period. A multidisciplinary team with experience in the telerehabilitation of pain- and stress-related exhaustion syndromes was another strength, allowing an existing clinical setting to be used to conduct the study, confirming the suitability for clinical implementation. The telerehabilitation approach was very much appreciated by participants living in different parts of Sweden, at that time being the only way for rehabilitation for the remaining symptoms after a SARS-CoV-19 acute infection.

Limitations of the study were as follows: (1) a limited sample size of the cohort, which was not sufficient to cover “drop-offs”, in particular those on the waiting list; (2) the sample’s sociodemographic composition; (3) limitations to fully control treatment adherence; (4) lacking to control placebo effects; (5) restriction to participants able to handle the Internet; (6) Swedish language; and (7) self-scored data as main outcomes. Since the study had an online approach for all moments, it can be seen both as a novelty and limitation. The absence of controlling medical recordings might be another limitation since all information was delivered by participants. In the spring of 2021, the National Board of Health and Welfare issued recommendations to primary healthcare providers regarding non-hospitalised patients with the post-COVID-19 condition, including offering rehabilitation (National Board of Health and Welfare, 2021). This may have prompted some on the waiting list to drop out of the study as they were keen to begin rehabilitation as soon as possible. Therefore, data of the six-month follow-up for both groups can include bias of other interventions in primary healthcare. Moreover, the above-mentioned limitations negatively impact the generalisability of this study and call for further studies.

The clinical application indicates several possibilities to develop and adapt multidisciplinary telerehabilitation to other patients’ populations having benefits for such interventions, especially groups having difficulties in attending physically due to health conditions, for example, chronic pain, including but not limited to headache/migraine where patients have difficulties attending physically due to attacks; fatigue conditions where patients experience difficulties in traveling due to limited energy, etc. Telerehabilitation may also solve the lack of interventions for people living in peripheral parts of the country. In this study, telerehabilitation was appreciated by participants from northern parts of Sweden and villages/cities far away from rehabilitation centers.

For cost-effectiveness, we are analysing the return-to-work data collected one year after telerehabilitation in comparison to those who did not participate due to different reasons, including non-randomised applicants. If cost-effectiveness is positive, the benefit of telerehabilitation should be reconsidered.

Conclusion: Our results show that multidisciplinary group telerehabilitation is beneficial for long COVID sufferers and improves health- and pain-related parameters, as well as emotional symptoms, after an eight-week programme and when followed up after six months. However, further research is needed to evaluate long-term “objective” data, such as socioeconomic costs for the intervention in order to understand the suitability in the clinical practice. Nevertheless, multidisciplinary telerehabilitation should be further developed and tested for other chronic conditions to prevent disability. For example, patients suffering from chronic pain and fatigue could be candidates for such interventions, particularly when the possibilities of physical rehabilitation are limited due to the condition by itself or the long distance to the health care providers.

## Figures and Tables

**Table 1:** Parameters for emotional status (HADS, PHQ-9, and GAD-7) as numbers of participants with abnormal values. ** indicates *p* < 0.01 between the telerehabilitation and waiting list.

Questionnaires	At Start N = 109	After Eight Weeks N = 109	After Six Months N = 81
HADS Anxiety			
All	16 (15%)	14 (13%)	7 (9%)
Telerehabilitation	11 (16%)	4 (6%) **	6 (10%)
Waiting list	5 (12%)	10 (24%)	1 (5%)
HADS Depression			
All	29 (27%)	12 (11%)	14 (17%)
Telerehabilitation	17 (25%)	8 (12%)	12 (20%)
Waiting list	12 (29%)	4 (10%)	2 (10%)
GAD-7			
All	17 (16%)	9 (8%)	9 (11%)
Telerehabilitation	11 (16%)	3 (5%)	9 (15%)
Waiting list	6 (14%)	6 (14%)	0
PHQ-9			
All	65 (60%)	46 (43%)	33 (43%)
Telerehabilitation	39 (59%)	26 (39%)	26 (43%)
Waiting list	26 (62%)	20 (48%)	10 (50%)

Abbreviations: HADS = Hospital Anxiety and Depression Scale; GAD-7 = (Generalised Anxiety Disorder), and PHQ-9 (Patient Health Questionnaire). Pathological values: HADS ≥ 11 (both anxiety and depression), PHQ-9 ≥ 10, and GAD-7 ≥ 10.

## Data Availability

The data that support the findings of this study are available from the first author (IBL) upon reasonable request and after completing approval by the Swedish Ethical Authorities (Etikprövningsmyndigheten).

## Supplementary Materials

The following supporting information can be downloaded at: https://www.mdpi.com/article/10.3390/jcm14020486/s1, Table S1: Number of participants indicating 36 IASP painful sites (multiple choices allowed) and only one most painful site, presented as the number of participants.
