# Supplementary material for: A First Randomized Eight-Week Multidisciplinary Telerehabilitation Study for the Post-COVID-19 Condition: Improvements in Health- and Pain-Related Parameters
Source: J Clin Med. 2025 Jan 14;14(2):486. doi: 10.3390/jcm14020486 (PMC11766284; doi:10.3390/jcm14020486)
Supplement: Supplementary file 1 [file jcm-14-00486-s001.zip › jcm-3310660-supplementary.pdf]

Supplemental Table S1. Number of participants indicating 36 IASP painful sites (multiple choices allowed) and only one most painful site presented as number of participants at the start, after eight weeks (8w) and at six months follow-up (6m).

**Sites**	**Left side, painful (start) N=109**	**Right side, painful (start) N=109**	**Left side, painful (8w) N=109**	**Right side painful (8w) N=109**	**Left side, painful (6m) N= 81**	**Right side, painful (6m) N=81**	**Most painful site in the body (start) N=109**	**Most painful site in the body (8w) N=109**	**Most painful site in the body (6m) N=81**
Head and/or face									
TR	68 (62%)	72 (66%)	64 (59%)	64 (59%)	54 (67%)	53 (65%)	34 (31%)	36 (33%)	31 (38%)
W	48 (72%)	48 (72%)	42 (63%)	27 (60%)	39 (65%)	38 (63%)	24 (36%)	24 (36%)	24 (36%)
Throat and/or neck									
TR	55 (51%)	52 (48%)	47 (43%)	48 (44%)	32 (40%)	34 (42%)	10 (9%)	8 (7%)	4 (5%)
W	34 (51%)	32 (48%)	30 (45%)	29 (43%)	21 (35%)	23 (38%)	6 (9%)	6 (9%)	6 (9%)
Shoulder									
TR	43 (39%)	35 (32%)	36 (33%)	38 (35%)	27 (33%)	28 (35%)	5 (5%)	8 (7%)	5 (6%)
W	26 (39%)	25 (38%)	23 (34%)	23 (34%)	18 (30%)	19 (32%)	2 (3%)	6 (9%)	6 (9%)
Upper arm							(for the shoulders,	(for the shoulders,	(for the shoulders,

TR<br>W	23 (21%)<br>15 (22%)	20 (18%)<br>16 (24%)	17 (16%)<br>13 (19%)	19 (17%)<br>14 (21%)	10 (12%)<br>7 (12%)	9 (11%)<br>6 (10%)	arms and hands)	arms and hands)	arms and hands)
Elbow<br>TR<br>WL	 12 (11%)<br>4 (16%)	 16 (15%)<br>6 (19%)	 9 (8%)<br>4 (6%)	 11 (10%)<br>5 (8%)	 10 (12%)<br>5 (8%)	 6 (7%)<br>4 (7%)			
Forearm<br>TR<br>WL	 17 (16%)<br>11 (16%)	 19 (17%)<br>13 (19%)	 17 (16%)<br>13 (19%)	 18 (17%)<br>13 (19%)	 6 (7%)<br>4 (7%)	 7 (9%)<br>5 (8%)			
Hand<br>TR<br>WL	 29 (27%)<br>15 (22%)	 28 (26%)<br>17 (25%)	 18 (17%)<br>8 (12%)	 21 (19%)<br>11 (16%)	 15 (15%)<br>9 (15%)	 17 (21%)<br>11 (18%)			
Front of the chest<br>TR<br>WL	 27 (43%)<br>31 (46%)	 33 (30%)<br>22 (33%)	 36 (33%)<br>22 (33%)	 32 (29%)<br>19 (28%)	 26 (32%)<br>21 (35%)	 21 (26%)<br>17 (28%)	14 (13%)<br>10 (15%)	10 (9%)<br>7 (10%)	10 (9%)<br>7 (9%)
Flank of the chest<br>TR<br>WL	 24 (22%)<br>14 (21%)	 16 (15%)<br>10 (15%)	 21 (19%)<br>14 (21%)	 21 (19%)<br>15 (22%)	 18 (22%)<br>13 (22%)	 12 (15%)<br>10 (17%)			

Abdomen									
TR	25 (23%)	25 (23%)	15 (14%)	16 (15%)	13 (16%)	15 (19%)	1 (1%)	2 (2%)	0
WL	14 (21%)	16 (24%)	8 (12%)	11 (16%)	9 (15%)	12 (20%)	0	1 (2%)	1 (2%)
Sexual organs									
TR	5 (5%)	5 (5%)	3 (3%)	3 (3%)	3 (4%)	3 (4%)	1 (1%)	1 (1%)	2 (3%)
WL	3 (5%)	4 (6%)	2 (3%)	2 (3%)	2 (3%)	2 (3%)	0	1 (2%)	1 (2%)
Thoracic spine									
TR	33 (30%)	34 (31%)	27 (25%)	23 (21%)	20 (25%)	17 (21%)	4 (4%)	4 (4%)	6 (7%)
WL	19 (28%)	19 (28%)	18 (27%)	14 (21%)	14 (23%)	14 (23%)	3 (5%)	4 (6%)	4 (6%)
Low back									
TR	38 (35%)	39 (36%)	36 (33%)	36 (33%)	25 (31%)	24 (30%)	5 (5%)	9 (8%)	3 (4%)
WL	23 (34%)	23 (34%)	22 (33%)	20 (30%)	17 (28%)	17 (28%)	1 (2%)	1 (2%)	1 (2%)
Hip and/or seat									
TR	27 (25%)	30 (28%)	27 (25%)	39 (28%)	24 (30%)	22 (27%)	7 (6%)	10 (9%)	4 (5%)
WL	14 (21%)	15 (22%)	16 (24%)	16 (24%)	15 (25%)	14 (23%)	6 (9%)	5 (8%)	5 (8%)
Thigh									
TR	20 (18%)	21 (19%)	20 (18%)	22 (20%)	10 (12%)	7 (9%)	20 (l8%)	11 (10%)	10 (12%)

WL	12 (18%)	12 (18%)	11 (16%)	12 (18%)	5 (8%)	4 (7%)	12 (18%) (for the entire legs)	5 (8%) (for the entire legs)	5 (8%) (for the entire legs)
Knee									
TR	14 (13%)	15 (14%)	17 (16%)	16 (15%)	13 (16%)	15 (19%)			
WL	8 (12%)	7 (10%)	9 (13%)	8 (12%)	9 (15%)	10 (17%)			
Lower legs									
TR	22 (20%)	24 (22%)	21 (19%)	21 (19%)	15 (19%)	12 (15%)			
WL	12 (18%)	12 (18%)	9 (13%)	9 (13%)	12 (20%)	10 (17%)			
Foot									
TR	27 (25%)	30 28%)	24 (22%)	23 (21%)	11 (14%)	15 (19%)			
WL	14 (21%)	16 (24%)	12 (18%)	12 (18%)	7 (12%)	10 (15%)			
No pain									
TR	9 (8.3%)	10 (9.2%)	14 (13%)	14 (13%)	7 (9%)	9 (11%)			
WL	5 (8%)	5 (8%)	10 (15%)	10 (15%)	5 (8%)	8 (13%)			
Pain sites varied									
TR							8 (7%)	10 (9%)	6 (7%)
WL							3 (5%)	7 (10%)	5 (8%)

Abbreviations: TR= telerehabilitation; WL=waiting list.
